# Annotations

**Published:** 1907-04-13

**Authors:** 


### Annotations.

### A Notable Character.

Mr. Thomas Henry Wakley, F.R.C.S., who died on the 5th instant in his 87th year, had been joint editor of the *Lancet* with his son, Mr. Thomas Wakley, Jun., since 1886. Mr. Wakley qualified nearly sixty years ago, and his active surgical work, as demonstrator to the late Sir Erasmus Wilson and as surgeon to the Royal Free Hospital, though belonging to a previous generation, brought him reputation and a large practice. On his father's death in 1862, the late Dr. James Wakley became editor, and Mr. Wakley manager of the *Lancet*. On the death of the former Mr. Wakley succeeded to the editorship. Having known Mr. Wakley for nearly forty years, and having been associated with him in journalism and in public work, the present writer can testify to his unostentatious but great liberality, to his amiable personality, and to his invariable desire to achieve results with the utmost possible consideration for the feelings and rights of other people. It is only a few months ago, in connection with a rather troublesome business affair, that Mr. Wakley displayed these admirable qualities and so contributed to the quiet settlement of a matter, which might otherwise have given rise to friction and difficulty. Mr. Wakley was one of the oldest and most respected members of the Council of the Metropolitan Hospital Sunday Fund, which became a metropolitan institution mainly through the influence of the *Lancet* and the persistent and continuous efforts of the late Dr. J. and Mr. Wakley. Though in no sense a public character, he had a host of friends who valued the qualities which so markedly characterised the man, and made him, often, a power for good in matters of importance. We offer our sincerest sympathy to Mrs. Wakley, and to Mr. Thomas Wakley also, who has shown such marked ability in the editorial chair of the *Lancet*, which is the oldest and probably the most influential medical journal in the world.

### Beri-Beri and Oxalic Acid Poisoning.

Eijkmann was the first to observe that by feeding fowls on rice they contracted a disease which closely resembled human beri-beri in its clinical features. Dyspnœa, cyanosis of the comb, and paralysis of the legs and wings were the chief symptoms. Maurer obtained similar results by feeding fowls with oxalic acid. Recently Dr. Adolphus Treutlein has confirmed and extended these observations. He fed fowls on oxalic acid, soluble oxalates and rice-meal, and obtained the same results in each case. On microscopic examination he found both in the heart and peripheral nerves a condition of fatty degeneration closely resembling the pathological lesions of human beri-beri. He attributes these changes to a separation of calcium from the tissues owing to the action of the oxalic acid introduced, or produced by decomposition of the rice-meal in the crop of the animal. By administering an excess of calcium carbonate in the food Dr. Treutlein was able to arrest the symptoms and check the pathological changes in the heart and peripheral nerves. In this way the oxalic acid was probably neutralised by the calcium and its deleterious effects prevented. In five cases of human beri-beri Dr. Treutlein found in the urine crystals of calcium oxalate far in excess of the amount present in normal urine. According to these results, the administration of calcium salts might also prove efficacious in human beri-beri and arrest the morbid process, at any rate in the early stages. Dr. Treutlein is hopeful that, owing to the close similarity of these morbid conditions in fowls to human beri-beri, the pathology of this disease may be elucidated by further researches on these lines, but it does not appear to have occurred to him to make practical use of the obvious therapeutic deductions. At any rate, there is no account of any attempt to test the value of calcium salts in the treatment of the disease.

### Chemical Research in Great Britain.

In his address as President of the Chemical Society, Professor Meldola gave an extremely gloomy and depressing account of the position and prospects of chemical research in Great Britain. He recognises that valuable work has been accomplished in such special institutions as the Royal Institution and the Davy-Faraday Laboratory, and that gratitude is due for results obtained by the Royal College of Science, the Central Technical College, and the Pharmaceutical Society. But, excluding these and some few other organisations, he finds little on which to erect a cheerful forecast. Our educational centres, such as universities and university colleges, judged by the continental standard, are pronounced to be distinct failures, and, with the exception of Manchester, it cannot be said that any one of them has developed an active school of chemical research. For this result, ancient traditions, defective educational methods, and want of sufficient means, leading to the frittering away of the research faculty by the drudgery of coaching, are held by Professor Meldola to be responsible. Nor can a more favourable verdict be passed on some modern educational organisations. Under Acts of Parliament passed in 1889 and 1890, large sums of money were placed at the disposal of county councils for the development of technical education, and though the primary object here was the improvement of technical skill, and knowledge of scientific principles among the artisan class, it was hoped that the new centres would offer to the appointed teachers opportunities for the cultivation of research work. The actual result is described as profoundly disappointing. And the failure is ascribed, not to the want of ability on the part of the teachers, but to the demands which are made on them to provide teaching for those wholly unprepared to receive it. Behind this, too, lies the entire want of appreciation of the importance of science and scientific research to the maintenance and development of the national welfare. This is the mental attitude of most popularly elected bodies in this country, and it cannot be doubted that, at least in this respect, the representatives accurately express the views of their constituents.

